# Composted Cattle Manure Increases Microbial Activity and Soil Fertility More Than Composted Swine Manure in a Submerged Rice Paddy

**DOI:** 10.3389/fmicb.2017.01702

**Published:** 2017-09-05

**Authors:** Suvendu Das, Seung Tak Jeong, Subhasis Das, Pil Joo Kim

**Affiliations:** ^1^Institute of Agriculture and Life Science, Gyeongsang National University Jinju, South Korea; ^2^Division of Applied Life Science (BK21+), Gyeongsang National University Jinju, South Korea; ^3^Division of Environmental and Industrial Biotechnology, The Energy and Resources Institute New Delhi, India

**Keywords:** livestock waste compost, soil enzyme, bacterial community, MiSeq, APIZYM

## Abstract

Livestock waste composts with minimum inorganic fertilizer as a soil amendment in low-input intensive farming are a feasible agricultural practice to improve soil fertility and productivity and to mitigate soil degradation. The key benefits of the practice rely on the activities of soil microorganisms. However, the role of different livestock composts [composted cattle manure (CCM) vs. composted swine manure (CSM)] on soil microbes, their activities and the overall impact on soil fertility and productivity in a flooded paddy remains elusive. This study compares the effectiveness of CCM and CSM amendment on bacterial communities, activities, nutrient availability, and crop yield in a flooded rice cropping system. We used deep 16S amplicon sequencing and soil enzyme activities to decipher bacterial communities and activities, respectively. Both CCM and CSM amendment significantly increased soil pH, nutrient availability (C, N, and P), microbial biomass, soil enzyme activities indicative for C and N cycles, aboveground plant biomass and grain yield. And the increase in above-mentioned parameters was more prominent in the CCM treatment compared to the CSM treatment. The CCM amendment increased species richness and stimulated copiotrophic microbial groups (Alphaproteobacteria, Betaproteobacteria, and Firmicutes) which are often involved in degradation of complex organic compounds. Moreover, some dominant species (e.g., *Azospirillum zeae*, *Azospirillum halopraeferens*, *Azospirillum rugosum*, *Clostridium alkalicellulosi*, *Clostridium caenicola*, *Clostridium termitidis*, *Clostridium cellulolyticum*, *Magnetospirillum magnetotacticum*, *Pleomorphomonas oryzae*, *Variovorax boronicumulans*, *Pseudomonas xanthomarina*, *Pseudomonas stutzeri*, and *Bacillus niacini*) which have key roles in plant growth promotion and/or lignocellulose degradation were enhanced under CCM treatment compared to CSM treatment. Multivariate analysis revealed that soil pH and available carbon (C) and nitrogen (N) were the major, while total organic carbon (TOC), total nitrogen (TN), and available phosphorus (P) were the minor drivers of variation in bacterial communities. Overall, our observations suggest that CCM amendment is better than CSM amendment to improve soil fertility and crop yield in a submerged rice cropping system.

## Introduction

A major challenge to modern intensive agriculture is to achieve high productivity while sustaining soil health and biodiversity. The intensive use of synthetic fertilizers, especially nitrogen (N) to achieve high yield often leads to soil degradation and acidification, which, in turn, deteriorates soil fertility and decreases crop yield (Ju et al., 2009). Low-input agricultural system which relies on the input of organic materials hold great promise not only to minimize the use of synthetic fertilizer, but also to improve crop productivity and to ensure ecosystem sustainability against nutrient mining and degradation of soil and water resources (Tilman et al., 2002; Kravchenko et al., 2017). Among the organic amendment, cattle and swine manure have been widely used in agricultural fields and the composted form of this manure is preferred over fresh manure to eliminate the risk of N loss via leaching and surface runoff, increase soil organic matter, suppress soil-borne pathogens and to mitigate greenhouse gas emissions (Darby et al., 2004; Evanylo et al., 2008; Kim et al., 2014; Escribano, 2016).

The livestock waste composts with minimum inorganic fertilizer as a soil amendment in low-input intensive farming has been well recognized as a vital agricultural practice to improve soil fertility and productivity (Tilman et al., 2002; Kravchenko et al., 2017). For instance, Francioli et al. (2016) reported that, compared to chemical fertilizer, the integrated use of chemical fertilizer (22.0% lower than the recommended dose) along with 20t ha^-1^ of farmyard manure (solid cattle manure with bedding) significantly (*p* < 0.05) increased soil organic matter, total nitrogen (TN) content and soil microbial biomass carbon (MBC) while crop yield was at par. Li et al. (2017) reported that the application of NPK + cattle manure and NPK + swine manure increased total organic carbon (TOC) by 143.4 and 54.7%, TN by 134.0 and 78.3% and crop yield by 48.9 and 39.6%, respectively, compared to NPK fertilization. Noteworthy, the key benefits of the practices rely on changes in soil microbial community and their activities which play an integral role in nutrient mobilization and plant growth promotion (Mader et al., 2002; Sun et al., 2015). In a literature review, Allison and Martiny (2008) reported that soil microbial community is mostly sensitive to nitrogen (N), phosphorus (P), and potassium (K) fertilization. Nitrogen is considered to be the major limiting nutrient for primary production in many terrestrial ecosystems and increased N input often leads to higher net primary production (Geisseler and Scow, 2014). While net primary production in terrestrial ecosystems, including rice paddies ecosystems, is generally N limited, soil microorganisms may be carbon (C) or N limited (Chen et al., 2014; Geisseler and Scow, 2014). The higher productivity brought on by fertilization in agricultural systems increases inputs of organic materials in the form of root exudates, decaying roots, and aboveground residues, and thus, increases the pool of C source for soil microorganisms (Geisseler and Scow, 2014; Hartmann et al., 2015). Although it has been well documented that both CCM and CSM improve soil fertility and productivity, our understanding of the soil microbial community influenced by CCM and CSM amendment is lacking. The differences in nutrient composition and indigenous microbial community in cattle and swine manure and their impact on the changes in soil physicochemical properties likely alter the soil microbial communities and activities. It has been reported that the indigenous microbiome of the livestock manure may not influence the soil microbial community as most of the bacterial species in livestock manure originate from the guts of animals and these are less competitive in the soil environment (Unc and Goss, 2004; Sun et al., 2015). Nonetheless, the use of livestock waste composts to manage the soil microbial community for the presence of beneficial and absence of detrimental microorganism may be promising to improve soil fertility and productivity (Hartmann et al., 2015).

Past studies revealed that the amendment of livestock waste composts either alone or in combination with inorganic fertilizer, improved enzyme activities and bacterial diversity in soil (Sun et al., 2015; Zhang et al., 2015). Sun et al. (2015) reported that long-term application of NPK chemical fertilizers caused a significant (*p* < 0.05) decrease of bacterial diversity, whereas addition of either swine manure or cattle manure restored bacterial diversity. Li et al. (2017) suggested that the combined application of organic (cattle manure compost) and inorganic (NPK) fertilization, not only increased soil organic carbon (SOC) and TN but also enhanced the bacterial community which is implicated in the decomposition of complex organic matter and soil carbon, nitrogen, and phosphorus transformations. While the effects of changes in nutrient availability due to fertilization on the soil microbial communities have received considerable attention, specific microbial taxa strongly influenced by cattle and swine manure compost fertilization and their down-stream influence on soil fertility and crop yield in flooded rice paddy systems needs further clarification.

Assuming that different livestock waste compost amendment has a different influence on soil nutrient availability and soil physicochemical properties, we hypothesized that these changes may strongly influence soil microbial community and their activities with positive cascade effects on soil fertility and productivity. Using a multidisciplinary approach that combined microbial community characterized by high-throughput sequencing and soil enzyme activities indicative of C, N, and P cycling, we aim to identify the most appropriate livestock waste compost amendment practice (CCM vs. CSM) to improve soil fertility and productivity in a flooded paddy. The objectives of our study were (i) to reveal the differences in soil bacterial communities in CCM and CSM amended soil, (ii) to assess the impact of different bacterial community on plant growth and C, N, and P acquisition, (iii) to explore the relationships between bacterial community composition and soil parameters.

## Materials and Methods

### Experimental Design and Sampling

The field experiment was conducted on Duryang experimental site (35^o^ 06’ N and 128^o^ 07’ E) of Gyeongsang National University, South Korea in 2014. The soil at the experimental site is classified as fine silty, mixed, mesic Typic Endoaquepts and have the following properties: pH (1:5 with H_2_O) 5.46 ± 0.4, organic carbon 16.16 ± 0.4 g kg^-1^, total N 1.22 ± 0.2 g kg^-1^, available P 57.8 ± 2.8 mg kg^-1^ and exchangeable K^+^, Ca^2+^, and Mg^2+^ 0.21 ± 0.03, 5.25 ± 0.20, and 0.51 ± 0.01 cmol^+^ kg^-1^, respectively.

Three treatments, i.e., control (C), composted cattle manure (CCM), and composted swine manure (CSM) were laid out in a split-plot design and replicated three times. The recommended rates of mineral fertilizers (N–P_2_O_5_–K_2_O = 90–45–58 kg ha^-1^) were applied (Rural Development Administration [RDA], 2010) in all treatments (including control). The mineral NPK fertilizers were applied in the form of urea, fused superphosphate and potassium, respectively. The basal mineral fertilizers applied 1 day before transplanting were 45 kg N ha^-1^, 45 kg P_2_O_5_ ha^-1^, and 40 kg K_2_O ha^-1^. Tillering fertilizer (22.5 kg N ha^-1^) was broadcasted about 2 weeks after rice transplanting and panicle fertilizer (22.5 kg N ha^-1^, 18 kg K_2_O ha^-1^) 6 weeks after transplanting. A day before the transplanting, composted cattle and swine manures was applied on an equal N basis (5 Mg ha^-1^ CCM and 6.35 Mg ha^-1^ CSM) in the respective fields. The composting process is described in the Supplementary Materials and the physicochemical properties of CCM and CSM are given in the Supplementary Table S1. Thirty days old rice (*Oryza sativa* Japonica L. *CV.* Dongjin) seedlings were transplanted and water level in all field plots was maintained at 5–7 cm throughout the experiment.

The soil samples were collected at the flowering stage of rice. The selection of the flowering stage for soil sampling is based on the fact that it is considered a key point of time for crop-induced changes in the soil microbial community since root growth and root exudation of rice reaches its peak near the flowering stage (Reichardt et al., 1997; Breidenbach et al., 2016). To study soil bacterial community, soil samples were collected from the rhizosphere region from each replicate plot and pooled into one composite sample. The rhizosphere soils were stored at -20°C until analysis. For soil chemical analysis and soil enzyme activities, eight soil cores (5 cm diameter and 20 cm depth) were randomly collected from each replicate plot and pooled into one composite sample. After root fragments and stones were removed, the soil was homogenized and passed through a 2 mm sieve. The freshly collected soil samples were used for the analysis of soil enzyme activities, whereas the air dried soil samples were used for soil chemical analysis.

### Soil Biochemical Analysis

Total organic C and TN in soil were measured according to Bremner and Mulvaney (1982) and Yeomans and Bremner (1988), respectively. The available P in soil was extracted with NaHCO_3_ and determined using the molybdenum blue method (Watanabe and Olsen, 1965). Exchangeable K^+^, Ca^2+^, and Mg^2+^ were extracted with 1 N NH_4_CH_3_CO_2_ (pH 7.0) and analyzed by atomic absorption spectrophotometer (Shimadzu 660, Kyoto). Readily mineralizable carbon (RMC) content and ninhydrin nitrogen content (NRN) which are indicative for available C and N, respectively (Inubushi et al., 1991), were estimated as reported by Das and Adhya (2014). MBC content of the soil was estimated by modified chloroform fumigation extraction method (Witt et al., 2000).

### Enzyme Fingerprinting Using APIZYM Assay

Soil enzyme activities indicative of C, N, and P cycling were measured using the semi-quantitative APIZYM system (Biomerieux, United States) as described by Martinez et al. (2016). The enzyme activities α-glucosidase, β-glucosidase, α-galactosidase, β-galactosidase, α-mannosidase, α-fucosidase, β-glucuronidase, esterase, lipase, and *N*-acetyl-β-glucosaminidase activities were measured to study C cycling. The enzyme activities aminopeptidase, e.g., leucine-aminopeptidase and cysteine-aminopeptidase, and protease, e.g., trypsin and chymotrypsin activities were measured to study N cycling. And the enzyme activities phosphohydrolase, acid and alkaline phosphomonoesterase activities were measured to study P cycling. Soil suspensions were prepared by adding 5.0 g of fresh soil in 50 ml of saline solution (0.85% NaCl) and homogenized in a horizontal shaker (IKA^®^ HS 501, United States) for 10 min. The soil suspensions were centrifuged at 2000 × *g* for 15 min and an aliquot (90 μl) of the supernatant was dispensed into microcupules containing different dehydrated chromogenic substrates for different enzymes (Supplementary Table S2). The APIZYM strips were covered and incubated at 30°C for 48 h followed by the addition of 30 μl of each reagent (ZYM A and ZYM B) to each microcupules to develop chromogenic substrates. After 5 min, color development was evaluated and a numerical value ranging from 1 to 5 (1 = 5 nM, 2 = 10 nM, 3 = 20 nM, 4 = 30 nM, and 5 = 40 nM) was assigned according to the color chart provided by the manufacturer. The results were reported as reactions of low intensity (0–1.9), moderate intensity (2–3.9), and high intensity (4–5) as described by Bonilla et al. (2015).

### DNA Extraction, PCR-Amplification, and Illumina Sequencing

The total genomic DNA from soil (0.8 g) was extracted using a PowerSoil^®^ DNA isolation kit (MO BIO, Carlsbad, CA, United States) according to the manufacturer’s instructions. PCR amplification was carried out using primer pair 515F (5′-GTGCCAGCMGCCGCGGTAA-3′) and 806R (5′-GGACTACHVGGGTWTCTAAT-3′) generating an about 280 bp amplicon from the variable V4 region of the 16S rRNA gene (Caporaso et al., 2012). Both primers contain Illumina adapter sequences while the reverse primer was barcoded with an 8-base sequence to facilitate multiplexing. PCR amplification was performed in 25 μl reactions containing 2.5 μl 10× AccuPrime PCR buffer II (including dNTPs) (Invitrogen, Grand Island, NY, United States), 0.4 μM of both forward and reverse primers, 10 ng template DNA, and 0.2 U AccuPrime High Fidelity Taq Polymerase. Triplicates of amplification were made for each sample and mixed after PCR amplification to minimize potential biases from amplification. Thermal cycling conditions were as follows: initial denaturation at 94°C for 1 min, followed by 30 cycles of 94°C for 15 s, 55°C for 15 s, 72°C for 30 s, and a final extension at 72°C for 10 min. PCR products were purified using a QIAquick PCR Purification kit (Qiagen) and quantified using Picogreen dsDNA assay (Invitrogen, United States). The purified amplicons were pooled in equimolar concentrations and loaded on a MiSeq Reagent Kit V2, and dual index sequencing of paired-end 250 bp was run on an Illumina MiSeq instrument (Illumina, San Diego, CA, United States). The 16S-rRNA gene sequences have been deposited in the GenBank short-read archive with accession number SRP101980 and under BioProject PRJNA379346.

### Bioinformatics and Statistical Analysis

Raw sequences were processed using the FastX Toolkit and the QIIME pipeline (Caporaso et al., 2012). Barcodes were recombined from paired-end reads, and forward reads were used for downstream analysis. All sequences were trimmed and low-quality sequences were removed. Quality filtering settings were as follows: minimum 30 quality scores over at least 75% of the sequence read; no ambiguous bases allowed; 1 primer mismatch allowed. Chimeras were further removed from the aligned 16S reads using a UCHIME algorithm (Edgar, 2010). The operational taxonomic units (OTUs) were identified based on 97% sequence similarity using a UCLUST algorithm (Edgar, 2010) in QIIME and the Greengenes database was used to assign taxonomy to each OTU (DeSantis et al., 2006). Additionally, we generated a species-level classification using ‘ClassifyReads’ a proprietary algorithm in MiSeq Reporter Metagenomics Workflow that provides species level classification for paired-end reads. This process involves matching short subsequences of the reads (called words) to a set of 16S reference sequences. The accumulated word matches for each read were used to assign reads to a particular taxonomic classification.

Permutational multivariate analysis of variances (PERMANOVA) and analysis of similarity (ANOSIM) was carried out to test the significant differences in bacterial community composition. The response ratio (RR) was employed to illustrate the changes in bacterial relative abundance (Shade et al., 2013). Mantel test, variation partitioning analysis (VPA) and canonical correspondence analysis (CCA) were performed to evaluate the linkages between the bacterial community and soil variables. To construct a CCA model, the predictor soil variables were selected based on their biological importance and *p*-values of single-factor CCA models and the variance inflation factor (VIF) criterion (VIF < 20). All the analyses were performed using functions in the Vegan package (v. 1.15-1) in R v. 2.8.1 (Oksanen et al., 2012). Estimates of α-diversity such as Shannon diversity index, Margalef’s Richness and Pielou’s Evenness were performed using Mothur (v.1.28.0). Univariate analysis of variance (ANOVA) followed by Tukey’s honestly significant difference (HSD) test was used to test differences in soil biochemical properties among the treatments.

## Results

### Soil Biochemical Properties

Each of the livestock waste composts (i.e., CCM and CSM) amendment significantly (*p* < 0.05) increased soil pH, TOC, TN, MBC, RMC, NRN, available P and exchangeable K^+^ as compared to the control (**Table 1**) and the increase was more prominent in the CCM treatment than the CSM treatment. Compared to CSM, CCM amendment significantly increased MBC by 15.8%, RMC by 14.7%, NRN by 7.5%, and available P by 6.8%. CCM amendment decreased C/N ratio compared to other treatments, however, the decrease was not significant.

**Table 1:** Soil biochemical properties and yield attributes under different fertilization regimes.

	Control	CCM	CSM
pH	5.46b	6.37a	6.10a
TOC (g kg^-1^)	16.16b	20.52a	18.52a
TN (g kg^-1^)	1.22b	1.60a	1.41a
C/N ratio	13.3a	12.8a	13.1a
MBC (mg kg^-1^)	337c	464a	401b
RMC (mg kg^-1^)	187c	273a	238b
NRN (mg kg^-1^)	5.52c	6.90a	6.42b
Available P (mg kg^-1^)	71.5c	103.8a	97.2b
*Exchangeable cation (cmol^+^ kg^-1^)*			
K^+^	0.26b	0.38a	0.34a
Ca^2+^	5.53a	5.63a	5.67a
Mg^2+^	0.53a	0.56a	0.54a
*Yield attributes*			
Grain yield (Mg ha^-1^)	6.30c	7.73a	7.18b
Straw yield (Mg ha^-1^)	8.13b	8.80a	8.67a
Total aboveground biomass (Mg ha^-1^)	14.43c	16.53a	15.90b
1,000 grain weight (g)	21.17b	23.27a	23.07a
Harvest index (%)	43.66c	46.78a	45.32b

*Values in the same row within the same parameters followed by different letters are significantly different at *p* < 0.05 according to Tukey’s HSD test. CCM, composted cattle manure; CSM, composted swine manure; TOC, total organic carbon; TN, total nitrogen; RMC, readily mineralizable carbon; NRN, ninhydrin nitrogen content; microbial biomass carbon. Harvest index is the ration of grain yield to the total aboveground biomass*.

### Plant Growth and Yield Attributes

The grain yield, straw yield, total aboveground biomass, 1,000 grain weight and harvest index were significantly (*p* < 0.05) increased by CCM and CSM amendment compared to that of the control treatment. Among the measured plant growth and yield attributes, grain yield, total aboveground biomass, and harvest index were significantly increased (*p* < 0.05) in CCM treatment over that of CSM treatment (**Table 1**).

### Soil Enzyme Activities

Each of the livestock waste composts induced an increase in enzyme activities responsible for C and N cycle and the increase was more prominent in the CCM treatment than the CSM treatment (**Table 2**). On the contrary, a decrease in enzyme activities responsible for P cycle was observed in CCM and CSM treatments compared to the control treatment. The highest overall enzyme activities were observed in CCM followed by CSM, control and bare soil (**Table 2**).

**Table 2:** Enzymatic profiles of soil based on the hydrolytic activities assessed by the APIZYM system.

	Enzyme	Bare soil	Control	CCM	CSM
C cycle	α-Glucosidase	0.7	1.3	1.7	1.7
	β-Glucosidase	1.0	1.7	4.3	3.7
	α-Galactosidase	0.7	1.3	2.7	2.3
	β-Galactosidase	2.3	2.7	4.7	4.0
	α-Mannosidase	0.3	0.7	1.3	1.3
	α-Fucosidase	0.3	0.3	1.0	0.7
	β-Glucuronidase	1.3	1.3	2.7	2.0
	Esterase	0.7	1.3	3.0	2.7
	Lipase	1.3	1.7	3.3	2.7
	*N*-Acetyl-β-glucosaminidase	1.7	2.3	3.3	2.7
N cycle	Leucine-aminopeptidase	1.3	1.3	4.0	2.7
	Cysteine-aminopeptidase	0.3	0.7	2.3	1.7
	Trypsin	0.3	0.7	2.0	1.0
	Chymotrypsin	0.0	0.3	1.3	0.7
P cycle	Phosphohydrolase	1.0	3.3	2.0	1.3
	Acid phosphomonoesterase	1.3	3.7	2.7	1.7
	Alkaline phosphomonoesterase	1.7	4.7	3.7	2.3
Total enzyme activities		16.3	29.3	46.0	34.3

*Values are the mean of three replicate the observations. Different font type indicates different intensities of the enzymatic activities: white, low intensity (0.0 to 1.9); light gray, moderate intensity (2.0 to 3.9); and dark gray, high intensity (4.0 to 5.0). CCM, composted cattle manure; CSM, composted swine manure*.

### Bacterial Community Structure

**FIGURE 1:**
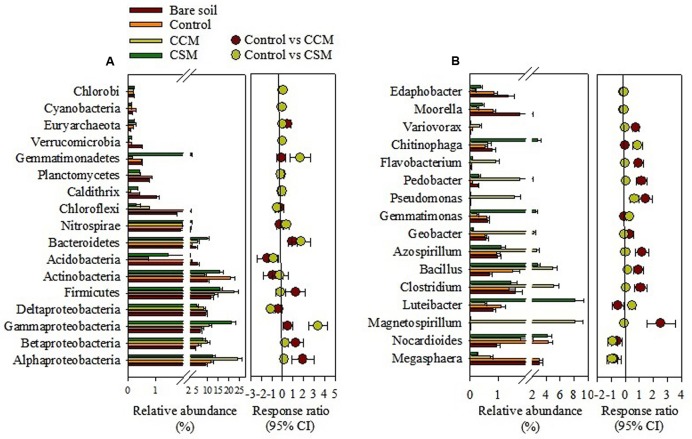
The relative abundance of major phylogenetic groups **(A)** and genera **(B)** in rhizosphere soil as influenced by fertilization. The relative abundance is presented in terms of percentage in total bacterial sequences per sample. Significantly altered phylogenetic groups and genera were presented in term of response ratio at 95% confidence interval. CCM, composted cattle manure; CSM, composted swine manure.

The phylum Proteobacteria dominated in the bare soil as well as in the fertilized soil, comprising 39.0, 39.2, 44.3, and 54.2% in the bare soil, control, CSM, and CCM treatments, respectively. Besides Proteobacteria, the other dominant phyla in the bare soil were Firmicutes (11.67%), Actinobacteria (9.8%), Acidobacteria (5.5%), and Bacteroidetes (4.8%), while in the control treatment they were Actinobacteria (20.7%), Firmicutes (13.5%), Bacteroidetes (2.7%), and Acidobacteria (2.4%) (**Figure 1** and Supplementary Table S3). In the CCM treatment, they were Firmicutes (22.4%), Actinobacteria (8.7%), Bacteroidetes (5.5%), and Acidobacteria (0.75%) and in the CSM treatment they were Firmicutes (15.8%), Actinobacteria (15.7%), Bacteroidetes (9.9%), and Acidobacteria (1.5%) (**Figure 1** and Supplementary Table S3). Significant increase in Alphaproteobacteria (*p* < 0.01), Betaproteobacteria (*p* < 0.05), Firmicutes (*p* < 0.01), and Bacteroidetes (*p* < 0.05) and decrease in Actinobacteria (*p* < 0.05) and Acidobacteria (*p* < 0.05) in the CCM treatment compared to the control was observed. Whereas, significant increase in Gammaproteobacteria (*p* < 0.01), Bacteroidetes (*p* < 0.01), and Gemmatimonadetes (*p* < 0.01) and decrease in Acidobacteria (*p* < 0.05) in the CSM treatment compared to the control treatment was recorded (Supplementary Table S3). Proteobacteria and Firmicutes increased by 22.4 and 41.5% in the CCM treatment compared to the CSM treatment, respectively. Among the Proteobacteria, 94.6 and 26.4% increase in Alphaproteobacteria and Betaproteobacteria in the CCM treatment compared to the CSM treatment was observed, respectively, whereas 48.2% decrease in Gammaproteobacteria in the CSM treatment compared to the CCM treatment was noticed.

The genus *Megasphaera* (comprising 3.1%) dominated in the bare soil, whereas *Nocardioides* (4.3%), *Magnetospirillum* (8.1%), and *Luteibacter* (8.1%) dominated in control, CCM, and CSM treatments, respectively. Other abundant genera in different treatments were shown in **Figure 1B**. CCM amendment induced a significant (*p* < 0.05) increase in the relative abundance of genera *Magnetospirillum*, *Clostridium*, *Bacillus*, *Azospirillum*, *Pseudomonas*, *Pedobacter*, *Flavobacterium*, and *Variovorax* compared to the control treatment (**Figure 1** and Supplementary Table S4). However, either no change or insignificant changes in the relative abundance of most of the genera (except *Luteibacter*) in the CSM treatment compared to that in the control treatment was noticed (**Figure 1** and Supplementary Table S4). Tremendous variation at the species level within the treatments was also observed. The species *Azospirillum zeae*, *Azospirillum halopraeferens*, *Azospirillum rugosum*, *Clostridium alkalicellulosi*, *Clostridium caenicola*, *Clostridium termitidis*, *Clostridium cellulolyticum*, *Magnetospirillum magnetotacticum*, *Pleomorphomonas oryzae*, *Pseudomonas xanthomarina*, *Pseudomonas stutzeri*, and *Bacillus niacini* which have a potential role in plant growth promotion and/or lignocelluloses degradation increased more in the CCM amendment than in control and CSM treatments (Supplementary Table S5).

The bacterial α-diversity estimation revealed that both of the livestock waste compost amendment significantly (*p* < 0.05) increased Margalef’s richness and Shannon Diversity indices compared to the control and the increase was more prominent in the CCM treatment than the CSM treatment. In contrast, a significant (*p* < 0.05) decrease in Pielou’s evenness in CCM and CSM treatments than the control treatment was observed (**Table 3**).

**Table 3:** The effects of livestock compost amendment on bacterial α-diversity indices.

Treatment	Margalef’s richness	Pielou’s evenness	Shannon Diversity
Bare soil	586c	0.86a	7.44b
Control	660b	0.85a	8.18ab
CCM	782a	0.80b	8.42a
CSM	766ab	0.82b	8.33a

*Mean of three replicates observations. In a column means followed by a common letter are not significantly different at *p* < 0.05. CCM, composted cattle manure; CSM, composted swine manure*.

### Linking Bacterial Community to Soil Variables

**FIGURE 2:**
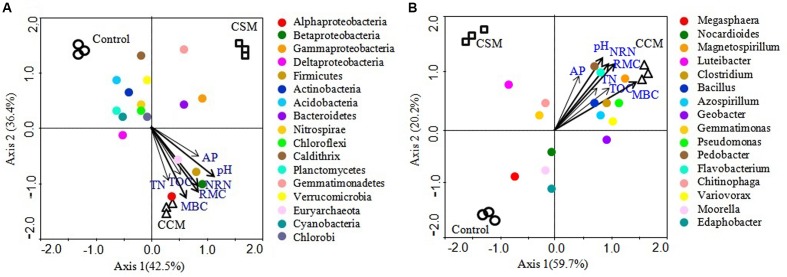
Canonical correspondence analysis (CCA) relating selected soil variables to major phylogenetic groups **(A)** and genera **(B)**. The resulting ordination biplot approximated the weighted average of each group/taxa with regard to each of the measured variables, which are represented as arrows. The lengths of these arrows indicate the relative importance of measured variables, whereas the angle between the arrows and the axis reflects the degree to which they are correlated. To statistically evaluate the significance (*p <* 0.01) of the first canonical axis and of all canonical axes together, the Monte Carlo permutation full model test with 999 unrestricted permutations was performed.

Mantel test analyses indicated that soil pH, TOC, TN, MBC, RMC, NRN, and AP were significantly correlated with soil bacterial communities (**Table 4**). Among the measured soil variables, soil pH, RMC, NRN, and MBC have the strongest influence on the bacterial communities. CCA showed that bacterial communities from different treatments were distinctly grouped and pH, TOC, TN, MBC, RMC, NRN, and AP were important soil attributes controlling bacterial community structure as they were significantly correlated with CCA axis 1 (*p* < 0.01) (**Figure 2**). Moreover, Alphaproteobacteria, Betaproteobacteria, Firmicutes, and Euryarchaeota were significantly and positively correlated while Actinobacteria, Acidobacteria, Chloroflexi, Planctomycetes, and Cyanobacteria were significantly and negatively correlated with pH, TOC, TN, MBC, RMC, NRN, and AP (**Figure 2** and Supplementary Table S6). The genera *Magnetospirillum*, *Clostridium*, *Bacillus*, *Azospirillum*, *Pseudomonas*, *Pedobacter*, *Flavobacterium*, and *Variovorax* were significantly and positively correlated, while *Megasphaera* significantly and negatively corrected with pH, TOC, TN, MBC, RMC, NRN, and AP (**Figure 2** and Supplementary Table S7). Partial CCA-based variation portioning analysis indicated soil variables, i.e., pH, TOC, TN, MBC, RMC, NRN, and AP explained 11.2, 8.3, 8.1, 12.0, 12.2, 11.9, and 7.6%, respectively, and their interaction explained 6.4% of the observed variance, while 28.7% of the variation was unexplained (*p* = 0.01).

**Table 4:** Correlation coefficient (*r*) between soil variables and bacterial community determined by Mantel test^†^.

Environmental variables (units)	*r*-value
Soil pH	0.52^∗∗^
TOC (g kg^-1^)	0.35^∗^
TN (g kg^-1^)	0.33^∗^
C/N ratio	-0.16 ns
RMC (mg kg^-1^)	0.54^∗∗^
NRN (mg kg^-1^)	0.48^∗∗^
Available P (mg kg^-1^)	0.31^∗^
Exchangeable K^+^ (cmol^+^ kg^-1^)	0.12 ns
Exchangeable Ca^2+^ (cmol^+^ kg^-1^)	0.06 ns
Exchangeable Mg^2+^ (cmol^+^ kg^-1^)	0.09 ns

^†^*Permutations, 9,999 (using the relative abundances of OTU as input in the analysis). ^∗∗^Significant at *p* < 0.01, ^∗^Significant at *p* < 0.05. RMC, readily mineralizable carbon; NRN, ninhydrin nitrogen content; TOC, total organic carbon; TN, total nitrogen*.

## Discussion

Although CCM and CSM amendment as an organic input in low-input intensive farming has been recognized as a highly valuable management practice to improve soil fertility and productivity, our understanding on which livestock compost is better to improve soil quality and productivity in rice cropping system is lacking. Our present study contributes toward understanding the effect of CCM and CSM amendment on soil fertility and productivity, with a focus on the response of bacterial communities in soil.

Our study revealed that CCM amendment was more effective in improving soil properties such as soil pH, TOC, TN, MBC, RMC, NRN, and available P than CSM with the same rate of application (**Table 1**). MBC is often used as an indicator of soil fertility since it responds promptly to soil changes (Brookes, 2001). The increase in MBC content in the CCM amended soil than the CSM amended soil could be attributed to the incorporation of easily degradable organic matter and other nutrients, which stimulates the growth of the autochthonous microorganisms of the soil. This is further supported by the increased species richness in the CCM amended soil than the CSM amended soil. The high availability of substrate in the CCM amended soil likely increased the species richness by promoting copiotrophic microorganisms, whose predominance, in turn, reduced evenness (Hartmann et al., 2015). The positive effect of microbial biomass by organic inputs has been well documented in rice cropping system (Liu et al., 2009; Nayak et al., 2012). The increase in C, N, and P availability and the buildup of microbial biomass in the CCM amended soil is expected to enhance productivity and indeed we observed significantly higher above ground biomass, grain yield, and harvest index in the CCM fertilized soil than the CSM fertilized soil.

Soil enzyme activities have been suggested as sensitive indicators of soil fertility since they catalyze the principal biochemical reactions (i.e., nutrient cycling, degradation of organic nutrient, and xenobiotics) that are essential for the maintenance of soil fertility (Nannipieri et al., 2012; Burns et al., 2013). The increased activities of C and N cycling enzymes in CCM and CSM amended soil compared to the control soil (**Table 2**) suggested that the CCM and CSM amendment would be a vital strategy to improve soil C and N turnover and fertility. The increased activities of these enzymes further indicated higher turnover rates of soil C and N and fertility in the CCM amended soil than that of CSM amended soil. The increased substrate availability and C demand (low TOC/TN ratio) for autochthonous microorganisms in the CCM amended soil could be the reason for higher C cycling enzyme activities in the CCM amended soil compared to the CSM amended soil. Enhanced C and N cycling enzyme activities as a consequence of livestock manure application have been reported in paddy soils (Zhang et al., 2015; Francioli et al., 2016). Interestingly, a decrease in P-cycling enzyme activities in both the livestock compost amended soil compared to the control soil was observed (**Table 2**). Reduced phosphatase enzyme activities as a consequence of the organic manure application have already been reported in agricultural soils (Francioli et al., 2016). The increase P demand for the soil microorganism may enhance the enzymatic acquisition of P in livestock amended soils.

While research has long focused on the effect of agricultural management on microbial biomass and soil enzyme activities, assessing microbial diversity has only recently become more accurate in the light of high throughput sequencing. One of the preeminent aspects of high throughput sequencing approach is the potential to identify the keystone species, influencing ecosystem functions (Hartmann et al., 2015). Proteobacteria and Firmicutes were more abundant in the CCM treatment compared to control and CSM treatments. Members of both phyla have been considered copiotrophs, i.e., organism that tends to grow faster in environments which are rich in nutrients, particularly carbon (Fierer et al., 2007). It is likely that the high availability of C, N, and P in the CCM treatment than other treatments, promoted the growth of the species belonging to these two phyla. A relatively higher abundance of Betaproteobacteria in the CCM treatment compared to the CSM treatment might be due to the greater carbon availability in the CCM treatment because Betaproteobacteria has been reported to respond quickly to carbon availability (Fierer et al., 2007; Das et al., 2016). Although, Alphaproteobacteria comprises bacteria with diverse physiological properties, the members of this class are well known for their role in N_2_-fixation (Lopes et al., 2014). With relatively high Alphaproteobacterial abundance, N_2_-fixation may substantially increase in the CCM treatment compared to control and CSM treatments. This was further supported by a noticeable increase in well known N_2_-fixing genera (*Azospirillum* and *Magnetospirillum*) in the CCM treatment than control and CSM treatments. The members of Firmicutes are generally considered as efficient degrader of lignocellulosic biomass and are consistently responded to organic amendment (Hartmann et al., 2015; Bonanomi et al., 2016). The significant increase in Firmicutes and the increase in the dominant genera, i.e., *Clostridium* and *Bacillus* in the CCM treatment compared to the CSM treatment, suggested that decomposition of lignocellulosic biomass might be favored by CCM amendment than CSM amendment. Besides *Clostridium* and *Bacillus*, *Pedobacter* and *Flavobacterium* which play a major role in lignocellulose degradation (Jimenez et al., 2016; Lopez-Mondejar et al., 2016) were significantly more abundant in the CCM treatment than in control and CSM treatments. Unlike Proteobacteria and Firmicutes, Acidobacteria exhibited much low abundance in the CCM treatment compared to control and CSM treatments. Notably, the relative abundance of Acidobacteria was much higher in the bare soil (**Figure 2** and Supplementary Table S2). Acidobacteria are generally considered as oligotrophs, i.e., an organism that can live in an environment that offers low levels of nutrients (Fierer et al., 2007). It is likely that the low nutrient availability promoted their growth in the bare soil while high nutrient availability diminished their growth in the CCM amended soil. Recent high throughput based studies reveal that Acidobacterial abundance decreased significantly with organic fertilizer amendment, including livestock manure amendment (Herzog et al., 2015; Sun et al., 2015; Francioli et al., 2016). Actinobacteria, which play a major role in the degradation of organochemicals (Bonanomi et al., 2016), were more abundant in the control treatment than CCM and CSM treatments. Some of the recent studies also showed a higher abundance of Actinobacteria in the conventional/NPK fertilized soil than the organic manure amended soil (Bonanomi et al., 2016; Francioli et al., 2016). The phyla Gemmatimonadetes and Verrucomicrobia were higher in abundance in the CSM treatment than the CCM treatment. The paucity of the cultured representative of these phyla makes it difficult to ascertain their probable role in the ecosystem. However, owing to their relatively high abundance and prompt response to the fertilizer management (Bergmann et al., 2011; Xuan et al., 2012), further research on their ecology and role in the environment is necessary.

Members of some dominant genera *Magnetospirillum*, *Azospirillum*, *Bacillus*, *Pseudomonas*, and *Variovorax* which have a key role in plant growth promotion (Supplementary Table S5) increased more in the CCM treatment than in control and CSM treatments. Identification of plant growth promoting bacteria at the lowest taxonomic (species) level was further explored because the taxonomy comparison at lowest level could be more appropriate to reveal differences (Shokralla et al., 2012). We found that some dominant species including *Azospirillum zeae*, *Magnetospirillum magnetotacticum*, *Pleomorphomonas oryzae*, *Azospirillum halopraeferens*, *Variovorax boronicumulans*, *Pseudomonas xanthomarina*, *Bacillus niacin*, *Pseudomonas stutzeri*, *Azospirillum rugosum*, and *Azospirillum picis* which have been reported as key players in plant growth promotion (Supplementary Table S5) were remarkably more abundant in the CCM treatment than other treatments; suggesting compared to the CSM amendment, the CCM amendment may enrich plant growth promoting bacteria in the flooded rice cropping system. The proliferation of plant growth promoting bacteria as a result of the organic amendment, including livestock manure amendment into the soil has been reported in several recent studies (Li et al., 2017; Zhang et al., 2017).

Soil properties play key roles in shaping microbial community structure and composition (Sun et al., 2015). In our study, from several soil variables, soil pH, available C (RMC), available N (NRN), and MBC were the major, while TOC, TN, and available P were the minor factors affecting the bacterial community structure. Noteworthy, compared to TOC and TN, available C and N had the strongest influence on the shift in soil bacterial community under livestock waste composts management in a flooded rice cropping system. The nutrient availability, particularly C and N and soil pH have been considered as the main driving factors for shift in soil microbial community under different fertilization regimes in different cropping systems, since these soil attributes may select some keystone species over others (Sun et al., 2015; Francioli et al., 2016).

Overall, our observation revealed that compared to CSM amendment, CCM amendment enhanced nutrient availability (mostly C, N, and P) and improved soil pH and in turn increased microbial biomass, species richness and promoted the proliferation of certain species which have key roles in the decomposition of complex organic matters and plant growth promotion. These changes, in turn, were eventually reflected in an enhanced aboveground biomass and grain yield under CCM amendment compared to CSM amendment in a flooded rice cropping system.

## Author Contributions

All authors contributed the intellectual input and assistance to this study and manuscript preparation. PK designed the research. SvD, SJ, and SbD conducted experiments and analyzed the data. SvD wrote the manuscript.

## Conflict of Interest Statement

The authors declare that the research was conducted in the absence of any commercial or financial relationships that could be construed as a potential conflict of interest.

## References

[B1] Allison S. D. Martiny J. B. H. (2008). Resistance, resilience, and redundancy in microbial communities. *Proc Natl Acad Sci U.S.A.* 105 11512–11519. 10.1073/pnas.0801925105 18695234 PMC2556421

[B2] Bergmann G. T. Bates S. T. Eilers K. G. Lauber C. L. Caporaso J. G. Walters W. A. (2011). The under-recognized dominance of verrucomicrobia in soil bacterial communities. *Soil Biol. Biochem.* 43 1450–1455. 10.1016/j.soilbio.2011.03.012 22267877 PMC3260529

[B3] Bonanomi G. Filippis F. D. Cesarano G. Storia A. L. Ercolini D. Scala F. (2016). Organic farming induces changes in soil microbiota that affect agroecosystem functions. *Soil Biol. Biochem.* 103 327–336. 10.1016/j.soilbio.2016.09.005

[B4] Bonilla N. Vida C. Martinez-Alonso M. Landa B. B. Gaju N. Cazorla F. M. (2015). Organic amendments to avocado crops induce suppressiveness and influence the composition and activity of soil microbial communities. *Appl. Environ. Microbiol.* 81 3405–3418. 10.1128/AEM.03787-14 25769825 PMC4407234

[B5] Breidenbach B. Pump J. Dumont M. G. (2016). Microbial community structure in the rhizosphere of rice plants. *Front. Microbiol.* 6:1537 10.3389/fmicb.2015.01537 PMC4710755 26793175

[B6] Bremner J. M. Mulvaney C. S. (1982). “Nitrogen-total,” in *Methods of Soil Analysis, Part2. Chemical and Microbiological Properties* Vol. 9 ed. Page A. L. (Madison, WI: American Society of Agronomy), 595–624.

[B7] Brookes P. (2001). The soil microbial biomass: Concept, measurement and applications in soil ecosystem research. *Microb. Environ.* 16 131–140.

[B8] Burns R. G. Deforest J. L. Marxsen J. Sinsabaugh R. L. Stromberger M. E. Wallenstein M. D. (2013). Soil enzymes in a changing environment: current knowledge and future directions. *Soil Biol. Biochem.* 58 216–234. 10.1016/j.soilbio.2012.11.009

[B9] Caporaso J. G. Lauber C. L. Walters W. A. Berg-Lyon D. Huntley J. Fierer N. (2012). Ultra-high-throughput microbial community analysis on the Illumina HiSeq and MiSeq platforms. *ISME J.* 6 1621–1624. 10.1038/ismej.2012.8 22402401 PMC3400413

[B10] Chen B. Liu E. K. Tian Q. Yan C. Zhang Y. (2014). Soil nitrogen dynamics and crop residues. A review. *Agron. Sustain. Dev.* 34 429–442. 10.1007/s13593-014-0207-8

[B11] Darby H. M. Stone A. G. Dick R. (2004). Compost and manure mediated impacts on soilborne pathogens and soil quality. *Soil Sci. Soc. Am. J.* 70 347–358. 10.2136/sssaj2004.0265

[B12] Das S. Adhya T. K. (2014). Effect of combine application of organic manure and inorganic fertilizer on methane and nitrous oxide emissions from a tropical flooded soil planted to rice. *Geoderma* 213 185–192. 10.1016/j.geoderma.2013.08.011

[B13] Das S. Chou M. L. Jean J. S. Liu C. C. Yang H. J. (2016). Water management impacts on arsenic behavior and rhizosphere bacterial communities and activities in a rice agro-ecosystem. *Sci. Tot. Environ.* 542 642–652. 10.1016/j.scitotenv.2015.10.122 26546760

[B14] DeSantis T. Z. Hugenholtz P. Larsen N. Rojas M. Brodie E. L. Keller K. (2006). Greengenes, a chimera-checked 16S rRNA gene database and workbench compatible with ARB. *Appl. Environ. Microb.* 72 5069–5072. 10.1128/AEM.03006-05 PMC1489311 16820507

[B15] Edgar R. C. (2010). Search and clustering orders of magnitude faster than BLAST. *Bioinformatics* 26 2460–2461. 10.1093/bioinformatics/btq461 20709691

[B16] Escribano A. J. (2016). “Organic livestock farming – challenges, perspectives, and strategies to increase its contribution to the agrifood system’s sustainability – a review,” in *Organic Farming – A Promising Way of Food Production*, ed. Konvalina P. (Rijeka: InTech). 10.5772/61272

[B17] Evanylo G. Sherony C. Spargo J. Starner D. Brosius M. Haering K. (2008). Soil and water environmental effects of fertilizer-, manure-, and compost-based fertility practices in an organic vegetable cropping system. *Agric. Ecosyst. Environ.* 127 50–58. 10.1016/j.agee.2008.02.014

[B18] Fierer N. Bradford M. A. Jackson R. B. (2007). Toward an ecological classification of soil bacteria. *Ecology* 88 1354–1364. 10.1890/05-1839 17601128

[B19] Francioli D. Schulz E. Lentendu G. Wubet T. Buscot F. Reitz T. (2016). Mineral vs. organic amendments: Microbial community structure, activity and abundance of agriculturally relevant microbes are driven by long-term fertilization strategies. *Front. Microbiol.* 7:1446 10.3389/fmicb.2016.01446 PMC5022044 27683576

[B20] Geisseler D. Scow K. M. (2014). Long-term effects of mineral fertilizers on soil microorganisms – A review. *Soil Biol. Biochem.* 75 54–63. 10.1016/j.soilbio.2014.03.023

[B21] Hartmann M. Frey B. Mayer J. Mader P. Widmer F. (2015). Distinct soil microbial diversity under long-term organic and conventional farming. *ISME J.* 9 1177–1194. 10.1038/ismej.2014.210 25350160 PMC4409162

[B22] Herzog S. Wemheuer F. Wemheuer B. Daniel R. (2015). Effects of fertilization and sampling time on composition and diversity of entire and active bacterial communities in german grassland soils. *PLoS ONE* 10:e0145575 10.1371/journal.pone.0145575 PMC4687936 26694644

[B23] Inubushi K. Brookes P. C. Jenkinson D. S. (1991). Soil microbial biomass C, N and ninhydrin-N in aerobic and anaerobic soils measured by fumigation-extraction method. *Soil Biol. Biochem.* 23 737–741. 10.1016/0038-0717(91)90143-8

[B24] Jimenez D. J. de-Lima Brossi M. J. Schuckel J. Kracun S. K. Willats W. G. T. van-Elsas J. D. (2016). Characterization of three plant biomass-degrading microbial consortia by metagenomics- and metasecretomics-based approaches. *Appl. Microbiol. Biotechnol.* 100 10463–10477. 10.1007/s00253-016-7713-3 27418359 PMC5119850

[B25] Ju X. T. Xing G. X. Chen X. P. Zhang S. L. Zhang L. J. Liu X. J. (2009). Reducing environmental risk by improving N management in intensive Chinese agricultural systems. *Proc. Natl. Acad. Sci. U.S.A.* 106 3041–3046. 10.1073/pnas.0813417106 19223587 PMC2644255

[B26] Kim S. Y. Pramanik P. Bodelier P. L. E. Kim P. J. (2014). Cattle manure enhances methanogens diversity and methane emissions compared to swine manure under rice paddy. *PLoS ONE* 9:e113593 10.1371/journal.pone.0113593 PMC4262209 25494364

[B27] Kravchenko A. N. Snapp S. S. Robertson G. P. (2017). Field-scale experiments reveal persistent yield gaps in low-input and organic cropping systems. *Proc. Natl. Acad. Sci. U.S.A.* 114 926–931. 10.1073/pnas.1612311114 28096409 PMC5293036

[B28] Li F. Chen L. Zhang J. Yin J. Huang S. (2017). Bacterial community structure after long-term organic and inorganic fertilization reveals important associations between soil nutrients and specific taxa involved in nutrient transformations. *Front. Microbiol.* 8:187 10.3389/fmicb.2017.00187 PMC5298992 28232824

[B29] Liu M. Hu F. Chen X. Huang Q. Jiao J. Zhang B. (2009). Organic amendments with reduced chemical fertilizer promote soil microbial development and nutrient availability in a subtropical paddy field: the influence of quantity, type and application time of organic amendments. *Appl. Soil Ecol.* 42 166–175. 10.1016/j.apsoil.2009.03.006

[B30] Lopes A. R. Manaia C. M. Nunes O. C. (2014). Bacterial community variations in an alfalfa - rice rotation system revealed by 16S rRNA gene 454-pyrosequencing. *FEMS Microbiol. Ecol.* 87 650–663. 10.1111/1574-6941.12253 24245591

[B31] Lopez-Mondejar R. Zuhlke D. Becher D. Riedel K. Baldrian P. (2016). Cellulose and hemicellulose decomposition by forest soil bacteria proceeds by the action of structurally variable enzymatic systems. *Sci. Rep.* 6:25279 10.1038/srep25279 PMC4850484 27125755

[B32] Mader P. Fliessbach A. Dubois D. Gunst L. Fried P. Niggli U. (2002). Soil fertility and biodiversity in organic farming. *Science* 296 1694–1697. 10.1126/science.1071148 12040197

[B33] Martinez D. Molina M. J. Sanchez J. Moscatelli M. C. Marinari S. (2016). API ZYM assay to evaluate enzyme fingerprinting and microbial functional diversity in relation to soil processes. *Biol. Fertil. Soils* 52 77–89. 10.1007/s00374-015-1055-7

[B34] Nannipieri P. Giagnoni L. Renella G. Puglisi E. Ceccanti B. Masciandaro G. (2012). Soil enzymology: classical and molecular approaches. *Biol. Fertil. Soils* 48 743–762. 10.1007/s00374-012-0723-0

[B35] Nayak A. K. Gangwar B. Shukla A. K. Mazumdar S. P. Kumar A. Raja R. (2012). Long-term effect of different integrated nutrient management on soil organic carbon and its fractions and sustainability of rice–wheat system in Indo Gangetic Plains of India. *Field Crops Res.* 127 129–139. 10.1016/j.fcr.2011.11.011

[B36] Oksanen J. Blanchet F. G. Kindt R. Legendre P. Minchin P. R. O’Hara R. B. (2012). Package ‘vegan’. *Commun. Ecol. Package* 2 1–291.

[B37] Reichardt W. Mascarina G. Padre A. Doll J. (1997). Microbial communities of continuously cropped, irrigated rice fields. *Appl. Environ. Microbiol.* 63 233–238. 16535489 10.1128/aem.63.1.233-238.1997 PMC1389103

[B38] Rural Development Administration [RDA] (2010). *Fertilization Standard of Crop Plants.* Suwon: Rural Development Administration.

[B39] Shade A. Caporaso J. G. Handelsman J. Knight R. Fierer N. (2013). A meta-analysis of changes in bacterial and archaeal communities with time. *ISME J.* 7 1493–1506. 10.1038/ismej.2013.54 23575374 PMC3721121

[B40] Shokralla S. Spall J. L. Gibson J. F. Hajibabaei M. (2012). Next-generation sequencing technologies for environmental DNA research. *Mol. Ecol.* 21 1794–1805. 10.1111/j.1365-294X.2012.05538.x 22486820

[B41] Sun R. Zhang X. X. Guo X. Wang D. Chu H. (2015). Bacterial diversity in soils subjected to long-term chemical fertilization can be more stably maintained with the addition of livestock manure than wheat straw. *Soil Biol. Biochem.* 88 9–18. 10.1016/j.soilbio.2015.05.007

[B42] Tilman D. Cassman K. G. Matson P. A. Naylor R. Polasky S. (2002). Agricultural sustainability and intensive production practices. *Nature* 418 671–677. 10.1038/nature01014 12167873

[B43] Unc A. Goss M. J. (2004). Transport of bacteria from manure and protection of water resources. *Appl. Soil Ecol.* 25 1–18. 10.1016/j.apsoil.2003.08.007

[B44] Watanabe F. S. Olsen S. R. (1965). Test of an ascorbic acid method for determining phosphorus in water and NaHCO3 extracts from soil. *Soil Sci. Soc. Am. J.* 29 677–678. 10.2136/sssaj1965.03615995002900060025x

[B45] Witt C. Gaunt J. L. Galcia C. C. Ottow J. C. G. Neue H. U. (2000). A rapid chloroform fumigation extraction method for measuring soil microbial biomass carbon and nitrogen in flooded rice soils. *Biol. Fertil. Soils* 30 510–519. 10.1007/s003740050030

[B46] Xuan D. Guong V. Rosling A. Alstrom S. Chai B. Hogberg N. (2012). Different crop rotation systems as drivers of change in soil bacterial community structure and yield of rice, *Oryza sativa. Biol. Fertil. Soils* 48 217–225. 10.1007/s00374-011-0618-5

[B47] Yeomans J. C. Bremner J. M. (1988). A rapid and precise method for routine determination of organic carbon in soil. *Commun. Soil. Sci. Plant.* 19 1467–1476. 10.1080/00103628809368027

[B48] Zhang Q. Zhou W. Liang G. Wang X. Sun J. He P. (2015). Effects of different organic manures on the biochemical and microbial characteristics of albic paddy soil in a short term experiment. *PLoS ONE* 10:e0124096 10.1371/journal.pone.0124096 PMC4399876 25879759

[B49] Zhang X. Zhang R. Gao J. Wang X. Fan F. Ma X. (2017). Thirty-one years of rice-rice-green manure rotations shape the rhizosphere microbial community and enrich beneficial bacteria. *Soil Biol. Biochem.* 104 208–217. 10.1016/j.soilbio.2016.10.023ref

