# Supplementary material for: Composted Cattle Manure Increases Microbial Activity and Soil Fertility More Than Composted Swine Manure in a Submerged Rice Paddy
Source: Front Microbiol. 2017 Sep 5;8:1702. doi: 10.3389/fmicb.2017.01702 (PMC5591829; doi:10.3389/fmicb.2017.01702)
Supplement: Supplementary file 1 [file Data_Sheet_1.PDF]

Supplementary materials

Composted cattle manure increases microbial activity and soil fertility more than composted swine manure in a submerged rice paddy

Suvendu Das [a]***, Seung Tak Jeong*** [b]***, Subhasis Das*** [c]***, Pil Joo Kim*** [a, b, *]

[a] *Institute of Agriculture and Life Science, Gyeongsang National University, Jinju 660-701, South Korea*

[b] *Division of Applied Life Science (BK 21+), Gyeongsang National University, Jinju 660-701, South Korea*

[c] *Division of Environmental and Industrial Biotechnology, The Energy and Resources Institute, New Delhi, India*

* Corresponding Author: Pil Joo Kim

Phone: +82-55-751-5466; Fax: +82-55-757-0178 14; E-mail: pjkim@gnu.ac.kr

Composting processes

The cattle and swine manures were collected from the nearby dairy farm and swine farm, respectively. A dump truck (170 kw, 62 kgm), a front loader (349 kw, 1800 rpm) and a turner (50 kw) were used to transfer the manures, to build up compost piles which are mixed with the fresh manure and the dried manure at a ratio of 8:5 (w/w) and to turn the piles once a day during the thermophilic stage. After thermophilic stage, the mixtures were stacked in the open-backyard for post-maturity. The composting experiment was conducted using a conventional static composting chamber method for 45 days in a greenhouse. The air-dried manure was filled in a plastic box 1.2 m^3 in size (1.2 m x 1.0 m x 1.0 m) with covering of styrofoam (5 cm thick) to preserve the heap temperature. The bio-oxidative phase of composting (active phase) was considered to be completed when the temperature of the pile was stable and close to the atmospheric temperature. The inner temperature of the pile was taken every day using thermometer inserted in the 3 sites at the middle part of the pile. Moisture levels of the piles were checked during the bio-oxidative phases of composting by taking representative samples from the 3 sites at the middle depth of the pile (40-50 cm). Pile was turned over 2 times (14th and 28th days) during the composting.

Table S1.Physicochemical properties of composted cattle manure (CCM) and composted swine manure (CSM)

Parameters	CCM	CSM
pH (1:5 with H_2O)	8.4±0.3	7.2±0.2
EC (dS m^{-1})	6.7±0.2	7.3±0.3
Total organic carbon (g C kg^{-1})	405±4.5	353±9.5
Total nitrogen (g N kg^{-1})	17.3±0.6	13.6±0.6
C/N ratio	23.4±0.8	25.9±1.2
Dissolved organic carbon (g C kg^{-1})	13.6±1.2	8.4±1.5
Dissolved organic nitrogen (g N kg^{-1})	3.2±0.12	1.9±0.08

Table S2. Overview of enzyme activities studied in soil using APIZYM assay.

	Process	Enzyme	EC number	Substrate
C cycle	Maltose degradation	α-glucosidase	3.2.1.31	2-naphthyl-2-D-glucopyranoside
	Cellobiose degradation	β-glucosidase	3.2.1.21	6-bromo-2-naphthol- α-D-galactopyranoside
	Melibiose degradation	α-galactosidase	3.9.1.1	6-Br-2-naphthyl- α-D-galactopyranoside
	Lactose degradation	β-galactosidase	3.2.1.22	2-naphthyl- α-D-galactopyranoside
	Mannose degradation	α-mannosidase	3.2.1.24	6-bromo-2-naphthyl-2-D-mannopyranoside
	Fructose degradation	α-fucosidase	3.2.1.51	2-naphthyl- αL-fucopyranoside
		β-glucuronidase	3.2.1.31	Naphthyl-AS-BI- βD-glucuronide
	Hemicelluloses degradation	Esterase	3.1.1.1	2-naphthyl-butyrate
		Lipase	3.1.1.3	2-naphthyl-myristate
	Polysaccharide degradation	N-acetyl-β-glucosaminidase	3.2.1.30	1-Naphthyl-N-acetyl-βD-glucosaminide
N cycle	N acquisition	Leucine-aminopeptidase	3.4.11.1	L-leucyl-2-naphthylamide
		Cystein-aminopeptidase	3.4.11.2	L-cystyl-2-naphthylamide
		Trypsin	3.4.11.3	N-benzol-DL-arginine-2-naphthylamide
		Chymotripsin	3.4.21.4	N-glutaryl-phenylalanine-2-naphthylamine
P cycle	P acquisition	Phosphohydrolase	3.1.3.2	Naphthyl AS-BI-phosphate
		Acid phosphomonoesterase	3.4.21.3	2 naphthyl-phosphate
		Alkaline phosphomonoesterase	3.1.3.1	2 naphthyl-phosphate

Table S3. The relative abundance of major bacterial phylotypes in rhizosphere soil as influenced by fertilization

	Bare soil	Control (NPK)	CCM	CSM
Alphaproteobacteria	9.06	11.71	24.14**	12.41
Betaproteobacteria	4.07	6.03	10.20*	8.07
Gammaproteobacteria	6.86	7.41	10.93	21.09**
Deltaproteobacteria	9.04	9.41	8.23	5.10
Firmicutes	11.67	13.54	22.38**	15.81
Actinobacteria	9.83	20.66	8.72*	15.73
Acidobacteria	5.51	2.39	0.75*	1.15*
Bacteroidetes	4.75	2.74	5.52*	9.90**
Nitrospirae	1.90	2.26	1.95	2.41
Chloroflexi	1.75	2.17	0.76	0.29
Caldithrix	1.03	0.43	0.11	0.35
Planctomycetes	0.78	0.86	0.44	0.41
Gemmatimonadetes	0.52	0.50	0.17	2.44**
Verrucomicrobia	0.53	0.12	0.05	0.13
Euryarchaeota	0.09	0.19	0.29	0.22
Cyanobacteria	0.16	0.28	0.15	0.14
Chlorobi	0.22	0.21	0.19	0.22

Data are presented as mean of triplicate observations. Differences were assessed by comparing CCM and CSM treatments with Control. * $p < 0.05$; ** $p < 0.01$.
CCM, composted cattle manure; CSM, composted swine manure.

Table S4. The relative abundance of major genera in rhizosphere soil as influenced by fertilization

Genera	Bare soil	Control (NPK)	CCM	CSM
Megasphaera	3.06	3.00	0.72	0.24*
Nocardioides	0.94	4.26	1.82	3.22
Magnetospirillum	0.03	0.03	8.08**	0.02
Luteibacter	0.81	1.11	0.52	8.14**
Clostridium	1.63	1.40	5.07**	1.46
Bacillus	0.69	1.54	4.90**	2.75
Azospirillum	0.98	0.94	2.57**	1.12
Geobacter	0.57	0.52	0.88	0.10
Gemmatimonas	0.62	0.60	0.26	0.94
Pseudomonas	0.02	0.02	1.80*	0.33
Pedobacter	0.28	0.08	1.59*	0.12
Flavobacterium	0.05	0.06	0.91*	0.07
Chitinophaga	0.79	0.54	0.15	1.89
Variovorax	0.01	0.07	0.86*	0.01
Moorella	1.79	0.81	0.26	0.44
Edaphobacter	1.38	0.86	0.18	0.37

Data are presented as mean of triplicate observations. Differences were assessed by comparing CCM and CSM treatments with Control. * $p < 0.05$; ** $p < 0.01$.
CCM, composted cattle manure; CSM, composted swine manure.

Table S5. Some of the dominant identified species in CCM treatment as compared to other treatments and their potential role

Species	Bare soil		Control		CCM		CSM		Role	Reference
	No. of sequences	Relative abundance (%)	No. of sequences	Relative abundance (%)	No. of sequences	Relative abundance (%)	No. of sequences	Relative abundance (%)		
Azospirillum zeae	20	0.007	142	0.04	5348	1.82	13	0.0004	N_2-fixation	Mehnaz et al., 2007
Azospirillum halopraeferens	547	0.22	1100	0.29	1799	0.61	91	0.03	N_2-fixation	Reinhold et al., 1987
Azospirillum rugosum	681	0.27	1103	0.29	1429	0.49	925	0.26	N_2-fixation	Lai et al., 2008
Clostridium alkalicellulosi	77	0.03	255	0.06	4503	1.54	236	0.07	Cellulose degradation	Zavarzin et al., 2008
Clostridium caenicola	98	0.03	76	0.02	2370	0.81	46	0.01	Cellulose/ cellobiose-degradation	Shiratori et al., 2009
Clostridium termitidis	4	0.001	16	0.004	1338	0.46	22	0.006	Cellulose degradation	Hethener et al., 1992
Clostridium cellulolyticum	2	0.0007	49	0.02	1140	0.40	42	0.01	Cellulose degradation	Desvaux, 2005
Magnetospirillum magnetotacticum	2	0.0007	7	0.001	5334	1.82	6	0.001	Siderophore production	Calugay et al., 2003
Pleomorphomonas oryzae	4	0.001	14	0.004	2110	0.72	0	0	N_2-fixation	Xie et al., 2005
Variovorax boronicumulans ‡	2	0.0007	12	0.003	1789	0.61	6	0.001	PGPR	Liu et al., 2013
Pseudomonas xanthomarina †	1	0.0003	1	0.0002	1603	0.55	2	0.0005	PGPR	Crovadore et al., 2016
Pseudomonas stutzeri	0	0	0	0	1522	0.52	4	0.001	N_2-fixation	Yan et al., 2008
Bacillus niacini	92	0.04	597	0.17	1570	0.54	849	0.24	PGPR	Kim et al., 2011

† Plant growth promoting rhizobacteria (PGPR), solubilize phosphate and produces indole-3-acetic acid, and siderophores.
‡ PGPR, produced a siderophore and phytohormone salicylic acid and degrade acrylamide
CCM, composted cattle manure; CSM, composted swine manure.

Table S6. Spearman correlation coefficients between the relative abundance of major phylogenetic groups and soil variables as determined by Mantel tests

	pH	TOC	TN	C/N ratio	MBC	RMC	NRN	AP	K^+	Ca^{2+}	Mg^{2+}
Alphaproteobacteria	0.61*	0.41*	0.40*	-0.33	0.65**	0.64**	0.57**	0.38*	0.19	0.06	0.11
Betaproteobacteria	0.66*	0.39*	0.37*	-0.33	0.61**	0.58**	0.56**	0.36*	0.15	0.13	0.04
Gammaproteobacteria	0.23	0.17	0.13	-0.11	0.23	0.18	0.18	0.32	0.02	0.05	-0.06
Deltaproteobacteria	-0.23	-0.25	-0.21	-0.13	-0.27	-0.21	-0.25	-0.19	-0.13	0.008	0.004
Firmicutes	0.56**	0.39*	0.38*	-0.33	0.58**	0.59**	0.55**	0.39*	0.11	0.08	0.03
Actinobacteria	-0.55**	-0.37*	-0.36*	0.32	-0.59*	-0.57**	-0.56**	-0.36*	-0.03	-0.11	-0.12
Acidobacteria	-0.63**	-0.36*	-0.33	0.31	-0.56**	-0.64**	-0.61**	-0.37*	-0.12	-0.06	-0.04
Bacteroidetes	0.18	0.23	0.13	-0.26	0.33	0.28	0.28	0.31	0.13	0.11	0.07
Nitrospirae	-0.16	-0.21	-0.17	0.18	-0.22	-0.21	-0.21	-0.26	-0.06	-0.05	-0.05
Chloroflexi	-0.39*	-0.36*	-0.35*	0.22	-0.36*	-0.37*	-0.39*	-0.36*	-0.19	-0.16	-0.03
Caldithrix	-0.18	-0.19	-0.09	0.23	-0.33	-0.33	-0.31	0.27	-0.06	-0.03	-0.04
Planctomycetes	-0.51**	0.39*	-0.37*	0.34	-0.53**	-0.57**	-0.55**	-0.38*	-0.23	-0.06	-0.09
Gemmatimonadetes	0.05	-0.13	-0.17	0.19	-0.15	0.07	0.07	0.27	0.01	0.06	-0.02
Verrucomicrobia	-0.26	-0.31	-0.29	0.23	-0.37*	-0.33	-0.33	0.30	0.16	0.15	0.13
Euryarchaeota	0.37*	0.39*	0.36*	-0.19	0.43*	0.57**	0.55**	0.36*	0.11	0.18	0.10
Cyanobacteria	-0.56**	-0.37*	-0.37*	0.25	-0.55**	-0.56**	-0.55**	-0.39*	-0.13	-0.08	-0.09
Chlorobi	-0.23	-0.26	-0.18	0.27	-0.25	-0.25	-0.26	-0.13	0.08	0.06	0.01

Significance: *, $p < 0.05$; **, $p < 0.01$.
MBC, microbial biomass carbon; RMC, readily mineralizable carbon; NRN, ninhydrin nitrogen content

Table S7. Spearman correlation coefficients between the relative abundance of major genera and soil variables as determined by Mantel tests

Genera	pH	TOC	TN	C/N ratio	MBC	RMC	NRN	AP	K^+	Ca^{2+}	Mg^{2+}
Megasphaera	-0.63**	-0.43*	-0.39*	0.29	-0.55**	-0.68**	-0.62*	-0.41*	-0.11	-0.13	-0.06
Nocardioides	-0.31	-0.30	-0.27	0.25	-0.28	-0.24	-0.21	0.26	-0.17	-0.13	-0.07
Magnetospirillum	0.66**	0.43*	0.39*	-0.19	0.65**	0.69**	0.57**	0.41*	0.18	0.17	0.21
Luteibacter	0.16	-0.02	-0.06	0.29	-0.04	0.04	0.10	0.29	0.08	0.07	0.09
Clostridium	0.57**	0.42*	0.41*	-0.26	0.63**	0.61**	0.60**	0.43*	0.19	0.21	0.05
Bacillus	0.61**	0.44*	0.37*	-0.28	0.66**	0.63**	0.59**	0.39*	0.14	0.12	0.19
Azospirillum	0.57**	0.43*	0.36*	-0.17	0.61**	0.60**	0.59**	0.37*	0.17	0.09	0.13
Geobacter	0.33	0.31	0.33	-0.23	0.27	0.26	0.23	0.17	0.15	0.13	0.20
Gemmatimonas	0.16	-0.13	-0.10	0.22	0.09	0.11	0.10	0.07	0.05	0.02	0.03
Pseudomonas	0.58**	0.42*	0.0.39*	-0.29	0.63**	0.61**	0.57**	0.38*	0.19	0.17	0.16
Pedobacter	0.61**	0.40*	0.0.39*	-0.23	0.66**	0.63**	0.61**	0.41*	0.22	0.04	0.06
Flavobacterium	0.57**	0.38*	0.39*	-0.18	0.59**	0.57**	0.55**	0.39*	0.26	0.07	0.13
Chitinophaga	0.26	0.13	0.09	0.27	0.16	0.23	0.21	0.17	0.03	0.03	0.05
Variovorax	0.37*	0.43*	0.37*	-0.21	0.46*	0.58**	0.40*	0.39*	0.08	0.03	0.06
Moorella	-0.29	-0.31	-0.26	0.22	-0.26	-0.27	-0.25	-0.19	-0.11	0.07	0.03
Edaphobacter	-0.28	-0.26	-0.25	0.23	-0.29	-0.26	-0.21	-0.31	-0.09	0.03	0.08

Significance: *, $p < 0.05$; **, $p < 0.01$.

References

Calugay, R.J., Miyashita, H., Okamura, Y., Matsunaga, T. (2003). Siderophore production by the magnetic bacterium *Magnetospirillum magneticum* AMB-1. *FEMS Microbiol. Lett.* 218(2), 371-5. doi:10.1016/S0378-1097(02)01188-6

Crovadore, J., Cochard, B., Calmin, G., Chablais, R., Schulz, T., and Lefort, F. (2016). Whole-genome sequence of *Pseudomonas xanthomarina* strain UASWS0955, a potential biological agent for agricultural and environmental uses. *Genome Announc*. 4(5), e01136-16. doi:10.1128/genomeA.01136-16. rançois

Desvaux**,** M. (2005). *Clostridium cellulolyticum*: model organism of mesophilic cellulolytic clostridia**.** *FEMS Microb. Rev*. 29(4), 741-764. doi:10.1016/j.femsre.2004.11.003

Hethener, P., Brauman, A., and Garcia, J.L. (1992). Clostridium termitidis sp. nov., a cellulolytic bacterium from the gut of the wood-feeding termite, *Nasutitermes lujae*. *System. Appl. Microbiol*. 15, 52-58.

Kim, W., Cho, W.K., Kim, S.N., Chu, H., Ryu, K.Y., Yun, J.C., and Park, C.S. (2011) Genetic diversity of cultivable plant growth-promoting rhizobacteria in Korea. *J. Microbiol. Biotechnol*. (2011), 21(8), 777-790. doi: 10.4014/jmb.1101.01031

Lai, W.A., Rekha, P.D., Arun, A.B., and Young, C.C. (2008). Effect of mineral fertilizer, pig manure, and *Azospirillum rugosum* on growth and nutrient contents of *Lactuca sativa* L. *Biol. Fertil. Soils*, 45, 155. doi:10.1007/s00374-008-0313-3

Liu, Z.H., Cao, Y.M., Zhou, Q.W., Guo, K., Ge, F., Hou, J.Y., Hu, S.Y., Yuan, S., and Dai, Y.J. (2013) Acrylamide biodegradation ability and plant growth promoting properties of *Variovorax boronicumulans* CGMCC 4969. *Biodegradation*. doi : 10.1007/s10532-013-9633-6

Mehnaz, S., Weselowski, B., and Lazarovits, G. (2007). Azospirillum zeae sp. nov., a diazotrophic bacterium isolated from rhizosphere soil of Zea mays. *Int. J. Syst. Evol. Microb*, 57, 2805–2809. doi: 10.1099/ijs.0.65128-0

Reinhold, B., Hurek, T., Fendrik, I., Pot, B., Gillis, M., Kersters, K., Thielemans, S., and De Ley, *Int. J. Syst. Evol. Microb*, 37, 43-51. Doi: 0020-77 13/87/0143-Q9$02.00/0

Shiratori, H., Sasaya, K., Ohiwa, H., Ikeno, H., Ayame, S., Kataoka, N., Miya, A., Beppu, T., and Ueda, K. (2009). *Clostridium clariflavum* sp. nov. and *Clostridium caenicola* sp. nov., moderately thermophilic, cellulose-/cellobiose-digesting bacteria isolated from methanogenic sludge. *Int. J. Syst. Evol. Microb*, 59, 1764–1770. Doi : 10.1099/ijs.0.003483-0

Xie, C.H., and Yokota, A. (2005). *Pleomorphomonas oryzae* gen. nov., sp. nov., a nitrogen-fixing bacterium isolated from paddy soil of Oryza sativa. *Int. J. Syst. Evol. Microb*, 55, 1233–1237. Doi : 10.1099/ijs.0.63406-0

Yan, Y., Yang, J., Dou, Y., Chen, M., Ping, S., Peng, J., Lu, W., Zhang, W., Yao, Z., Li, H., Liu, W., He, S., Geng, L., Zhang, X., Yang, F., Yu, H., Zhan, Y., Li, D., Lin, Z., Wang, Y., Elmerich, C., Lin, M., and Jin, Q. (2008). Nitrogen fixation island and rhizosphere competence traits in the genome of root-associated *Pseudomonas stutzeri* A1501. Proc. Natl. Acad. Sci. USA, 27, 105(21), 7564–7569. doi: 10.1073/pnas.0801093105

Zavarzin, G.A., Zhilina, T.N., and Dulov, L.E. (2008). Alkaliphilic sulfidogenesis on cellulose by combined cultures. *Microbiol*. 77(4), 419-429. doi:10.1134/S0026261708040061
